# Mobile Phone Addiction and Suicidal Behaviors in Adolescents: School-Based Cross-Sectional Study in Zhejiang Province, China

**DOI:** 10.2196/80410

**Published:** 2025-11-24

**Authors:** Zhecong Yu, Zesheng Chen, Yaoyao Wu, Zongxue Cheng, Biao Li, Caixia Jiang, Jue Xu, Meng Wang

**Affiliations:** 1 Hangzhou Center for Disease Control and Prevention (Hangzhou Health Supervision Institution) Hangzhou, Zhejiang China; 2 Zhejiang Provincial Center for Disease Control and Prevention Hangzhou, Zhejiang China

**Keywords:** adolescents, mobile phone use, suicidal behaviors, mediation analysis, cross-sectional study

## Abstract

**Background:**

Suicide is a critical public health issue in adolescents. Mobile phone addiction (MPA) is characterized by an impaired ability to use mobile phones appropriately, which may further induce addictive symptoms analogous to those of substance abuse disorders and exhibit a significant association with suicidal behaviors. However, the associations between MPA and suicidal behaviors have been inconsistent across previous studies in adolescents.

**Objective:**

This study aimed to investigate the association between MPA and suicidal behaviors and to explore the potential mediating roles of sleep quality, depression, and anxiety.

**Methods:**

A total of 27,070 adolescents aged 10-19 years were enrolled from a cross-sectional survey with a multistage cluster sampling method across 376 schools in Zhejiang Province, China. A total of 26,394 adolescents with complete data were included in the final analysis. MPA assessed by the Mobile Phone Addiction Index, mobile phone ownership, suicidal behaviors (including suicide ideation, plans, and attempts), sleep duration, depression assessed by Patient Health Questionnaire 9-Item Scale, and anxiety assessed by Generalized Anxiety Disorder 7-Item Scale were obtained from a self-administered questionnaire. Multinomial logistic regression was used to examine the associations of mobile phone ownership and MPA with suicide behaviors. Multivariate linear regression was used to assess the potential mediating effect of sleep duration, depression, and anxiety on these associations.

**Results:**

MPA was identified in 4350 (16.5%) adolescents, and 22,299 (84.5%) reported mobile phone ownership. A history of suicidal ideation was reported by 3187 (12.1%) adolescents, suicide plans by 1378 (5.2%), and suicide attempts by 538 (2%). After adjusting for potential confounders, MPA was significantly associated with increased odds of suicide ideation (odds ratio [OR] 2.449, 95% CI 2.236-2.683) and suicide attempts (OR 3.934, 95% CI 3.263-4.743) compared to the normal. Compared to the suicide ideations, MPA had a 1.45 times higher odds of having suicide plans (OR 1.453, 95% CI 1.265-1.669). Compared to adolescents who had neither mobile phone ownership nor MPA, MPA without mobile phone ownership had significantly higher suicidal scores (β coefficient=0.498, 95% CI 0.407-0.588). Mediation analysis indicated that sleep duration (β coefficient=–0.055, 95% CI –0.060 to –0.050), depression (β coefficient=0.952, 95% CI 0.927-0.977), and anxiety (β coefficient=1.141, 95% CI 1.130-1.152) partially mediated the association between MPA and suicidality.

**Conclusions:**

MPA was dependently associated with suicidal behaviors in adolescents, with sleep duration, depression, and anxiety partially mediating this association. MPA without mobile phone ownership was related to a higher suicidal score. These findings highlight the importance of addressing the issues of mobile phone use for suicide prevention among Chinese adolescents.

## Introduction

Suicide is a serious public health issue. Globally, ~100,000 suicide cases are reported annually in young people aged 15-29 years, with the fourth leading cause of death in adolescents [1]. Notably, in the United States, the annual rate of adolescent suicides increased by 3.1% between 2007 and 2014 [2], whereas in China, suicide is the leading cause of death among youths aged 15-34 years [3]. A substantial increase has been noted in children aged 5-14 years and adolescents aged 15-24 years since 2017 [4]. Against the backdrop of the global and regional burden of suicide, emerging risk factors associated with the digital era have increasingly emerged as critical to understanding adolescent suicide—traditional biological, psychological, and social factors are increasingly insufficient to fully account for the escalating suicide rate [5], and the potential association between problematic digital technology use (eg, problematic mobile phone use) and suicidal behaviors has garnered growing scholarly interest [6]. Suicidal behavior is a complex and continuous process involving suicide ideation, suicide plans, suicide attempts, and completed suicide [7]. Nonfatal suicidal behaviors are strongly linked to later suicide. Evidence showed that approximately one-third with suicide ideation went on to develop a suicide plan, and approximately two-thirds of those with a plan attempted suicide [8]. Targeted prevention efforts are recommended for high-risk populations such as children and adolescents [4].

The proliferation of the internet has made mobile phones indispensable in modern life, but this digital transformation also brings unique mental health challenges for adolescents. Mobile phones can provide convenient and accessible means for their escapism (a behavior characterized by excessive engagement in an activity to cope with difficult life situations from reality), which may exacerbate problematic mobile phone use [9], and adolescents were more susceptible than adults since they lack a stable ability to regulate emotions and manage behaviors in the digital age [10-12]. Problematic mobile phone use in adolescents was significantly related to the odds of suicide ideation and suicide attempts compared to those with general mobile phone use [13]. Approximately 90% of minor internet users have personal internet devices, and approximately two-thirds of them have mobile phone ownership [14]. Meanwhile, the prevalence of mobile phone addiction (MPA) among youths is rapidly rising [15]. MPA or problematic mobile phone use refers to the loss of the ability to use mobile phones properly, which can further lead to addictive symptoms similar to substance abuse disorders, including compulsive use, loss of control, withdrawal, craving, and mood dysregulation [15].

While previous studies across different countries have consistently identified a significant association between MPA and suicidal behaviors, the specific relationships between MPA and suicidal ideation, plans, or attempts remained inconsistent [16-19]. Notably, the methods for estimating the prevalence of MPA and suicidal behaviors included multistage cluster sampling with large sample sizes or convenience sampling with small sample sizes, and the assessment of MPA used different tools, including the Mobile Phone Addiction Index (MPAI), Smartphone Addiction Scale-Short Version, or self-designed questionnaires. Measurement heterogeneity, along with differences in cultural backgrounds and demographic characteristics, should be taken into account [16,20,21]. In addition, few studies focused on the interaction between mobile phone ownership and MPA [22]. Mobile phone ownership enabled escapism to occur at any time, which may facilitate addiction, while the absence of a mobile phone may trigger “nomophobia” [23]. It remained unclear whether its interaction affects suicidal behaviors in adolescents. Importantly, physiological and psychological factors often serve as a “bridge,” linking external factors to suicidal behaviors [24,25]. A meta-analysis showed that poor sleep quality, depression, and anxiety were adverse outcomes of MPA [26]. Cross-sectional studies of adolescents in China found that poor sleep quality, depression, and anxiety were positively correlated with the prevalence of suicide [27,28]. Cheng et al [16] and Chen et al [17] found that limited mobile phone use, good sleep quality, and preventing depression could be practical for keeping adolescents from suicide behaviors. To our knowledge, studies detailing the mediating roles of these factors in the association between MPA and suicidal behaviors among a large sample of Chinese adolescents are rather limited.

Therefore, this study aimed to explore the associations of mobile phone use with suicidal behaviors among Chinese adolescents. Besides, the mediating roles of sleep duration, depression, and anxiety in these investigated associations were also evaluated.

## Methods

### Study Design and Participants

**Figure 1:**
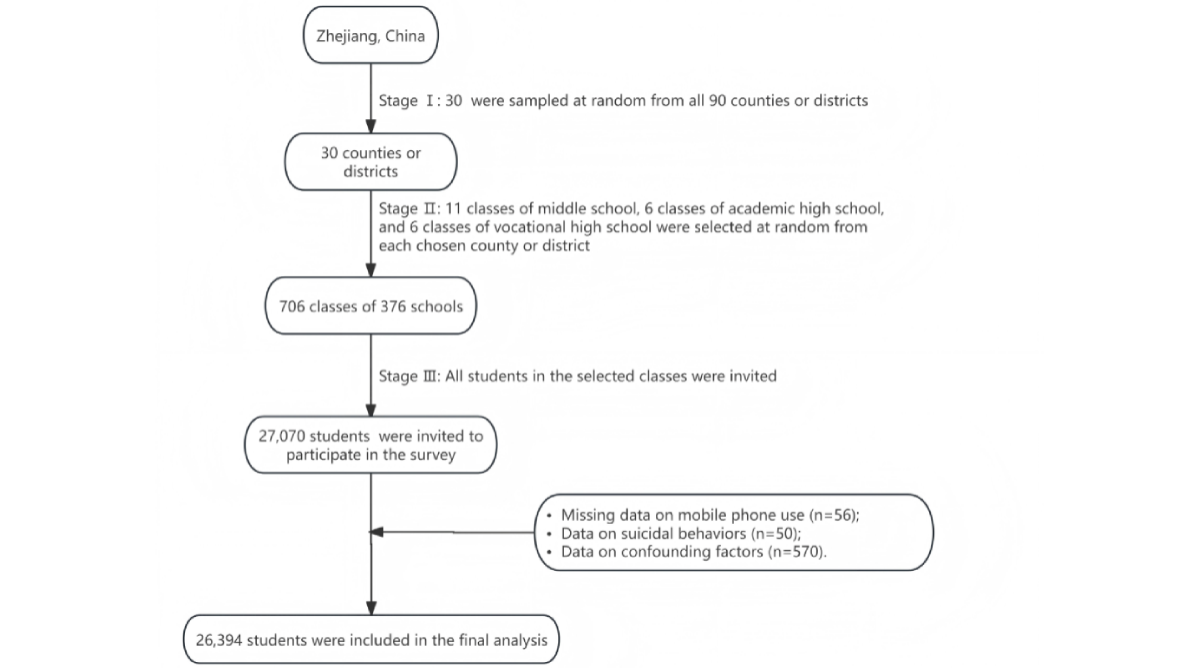
A flowchart of the study population with a multistage cluster sampling design.

In March 2022, the Zhejiang Youth Risk Behavior Survey of China, a school-based cross-sectional study, was conducted by the Zhejiang Provincial Center for Disease Control and Prevention. Detailed methodology has been described previously [29]. Information on health-related behaviors of students was collected using self-reported questionnaires adapted from the US Youth Risk Behavior Surveillance System and the Global School-Based Student Health Survey. Students completed the anonymous questionnaire in an unsupervised classroom setting, seated at spaced intervals to prevent them from viewing each other’s responses. Their answers were irrelevant to their academic performance and would be kept confidential. The completed questionnaires were placed by the students into a sealed box. Students with serious health conditions or illnesses, including intellectual disability or language disorder, were excluded from the survey. Finally, a large representative random sample comprising students from middle, academic, and vocational high schools was selected using a multistage cluster sampling design. For this analysis, 27,070 middle and high school students from 706 classes of 376 schools in 30 counties meeting the following criteria were excluded: (1) missing data on mobile phone use (n=56), (2) missing data on suicidal behaviors (n=50), and (3) missing data on confounding factors (n=570). This resulted in a final analytical sample of 26,394 participants (Figure 1).

### Data Collection and Definitions

Demographic variables (eg, school types, gender, age, ethnicity, and parental educational levels), lifestyle factors (smoking and drinking), and physical indicators (weight and height) were obtained as confounding factors from a questionnaire. Smoking was defined as having smoked cigarettes on at least 1 day in the past 30 days. Drinking was defined as having consumed alcohol on at least 1 day in the past 30 days. Wake lag and bed lag were assessed using the following questions: “How much later do you wake up or go to bed on weekends compared to schooldays in the past 30 days?” Sleep duration was assessed by the question: “During the past 30 days, how long (hours and minutes) did you sleep every day on average (including daytime rest)?” and was calculated as: hours+(minutes/60). Depressive symptoms were assessed using the Chinese version of the Patient Health Questionnaire 9-Item Scale (PHQ-9) [30]. Participants were asked to report the presence of 9 problems, including depression and interest decline. Each item was rated on a 4-point Likert-type scale ranging from 0=not at all to 3=nearly every day. Total score ranged from 0 to 27 points, with higher scores indicating poorer depressive symptom performance. The PHQ-9 scale has demonstrated good reliability and validity in Chinese adolescents, with a Cronbach α of 0.86 [31]. Anxiety symptoms were assessed using the Chinese version of the Generalized Anxiety Disorder 7-Item Scale (GAD-7) [32]. In brief, each item is rated on a 4-point Likert-type scale ranging from 0=not at all to 3=nearly every day. Total score ranged from 0 to 21 points, with higher scores indicating better anxiety symptom performance. GAD-7 has been validated among the adolescent population in China, with a Cronbach α of 0.94 [33].

The measurement of mobile phone use included 2 dimensions: mobile phone ownership and MPA. Mobile phone ownership was assessed by asking the following question: Do you have your mobile phone ownership (Yes or No)? MPA was assessed using the Chinese version of the MPAI [34]. MPAI has been well-validated in Chinese adolescents, with the Cronbach α of all its subscales being>0.72 [35]. The MPAI consists of 17 items, with each item scored on a 5-point Likert-type scale ranging from 1=not at all to 5=always, including losing control and receiving complaints, anxiety and cravings, withdrawal or escape, and productivity loss. The total score of MPAI ranged from 17 to 85, with higher scores indicating a higher intensity of mobile phone use. In this study, MPA was defined as a score of 51 or above [36].

Suicidal behaviors was measured by using the following 3 items, according to the Global School-Based Student Health Survey [37], including suicide ideation, plans, and attempts, respectively: “Have you seriously considered suicide in the last 12 months?” “Have you made a specific plan to commit suicide in the last 12 months?” and “Have you attempted suicide in the last 12 months?” Given that suicidal behaviors represent a developmental process [38], a skip logic was implemented for these items. The detailed classification process is present in Multimedia Appendix 1. To observe the relationship between MPAI and the evolution process of suicide behaviors, we added 1 item about hospital visits for suicidal behaviors, which captured the severity of the behavior post occurrence [39]. Suicidal behavior score was calculated, with an equidistant transformation (1-point increment per category) applied to convert this score into a continuous variable [17]. Total score ranged from 4 to 10 points, with higher scores indicating more severe suicidal behaviors. The scoring guideline is present in Multimedia Appendix 2.

### Statistical Analysis

Continuous variables were presented as mean and SD, and categorical variables were expressed as numbers and percentages (%). Values between gender groups were compared using variance (ANOVA) or independent 2-tailed *t* test and chi-square test or Fisher exact test, as appropriate. To evaluate the associations of mobile phone ownership and MPA with suicidal behaviors, multinomial logistic regression analysis was used to calculate the odds ratios (ORs) with corresponding 95% CIs. Two models were established: model 1 was unadjusted. Model 2 was adjusted for gender, age, ethnicity, registered permanent residence, school types, parents’ education level, parents’ marital status, single child, family economic status, accommodation, height, weight, smoking, and drinking. β coefficients were also calculated with a multivariate linear regression model entering suicidal scores as the dependent variable and were adjusted for the same variables as model 2. Restricted cubic splines with 3 knots at the 25th, 50th, and 75th percentiles were performed to flexibly model the association of MPAI and suicidal scores based on linear regressions and the association between MPAI and suicidal behaviors based on binary logistic regressions.

Subgroup analysis was stratified by gender, age, school types, accommodation, parents’ marital status, single child, family economic status, and overweight and summarized with forest plots. An interaction term within each subgroup was calculated. Specifically, the cutoff point for the age group was 14 years [40], and the overweight group was BMI>mean+1SD [41]. For sensitivity analysis, we further adjusted model 2 with adding mobile phone placement habits and its use duration on schooldays and weekends. Besides, given that some participant characteristics were significantly different in participants, propensity score weighting (PSW) using inverse probability weight was calculated, and PSW multinomial logistic regression models were established to further control confounders [42]. To verify the robustness of the relationship between MPA and suicidal behaviors, we would redefine MPA using a cutoff of MPAI≥60 and repeat the analysis adjusted in accordance with model 2 [43]. We finally used the *E*-value as a measure to evaluate the stability of linear regression models. This metric aids in assessing whether an unmeasured underlying factor may influence an observed link. Mediation analysis was carried out via the multivariate linear regression with a bootstrapping method (a bootstrap sample with 5000 was performed) to investigate the potential mediating effects of sleep duration, depression, and anxiety (mediators) on the associations between MPAI (exposure) and suicidal scores (outcome). We calculated the predicted values of the multivariate linear regression model with the adjusted model 2 for sleep duration, depression, anxiety, MPAI, and suicidal scores and entered them into the mediation pathways, respectively. We defined three pathways: (1) exposure to mediator, (2) mediator to outcome, and (3) exposure to outcome. The total effect was obtained through the sum of a direct effect and a mediated (indirect) effect. The percentage of the mediated effect was calculated using the formula: mediated effect/total effect×100%.

Data analysis was performed using the SAS software (version 9.4; SAS Institute Inc). The mediation analysis was performed using AMOS (version 23.0; IBM Corp). A 2-tailed *P* value of <.05 was accepted as indicating statistical significance.

### Ethical Considerations

The study complied with the Declaration of Helsinki and the Guidelines for Primary and Secondary School Science Education Work [44] and was approved by the ethics committee of Zhejiang Provincial Center for Disease Control and Prevention (approval code: 2022-007-01). Before the survey was conducted, all parents or legal guardians received a hard copy of the “Notice to Parents.” This notice included a detailed explanation of the survey and an authorized section for handwritten signatures to consent their child’s participation in this survey. Written informed consent was obtained from all students with the assistance of the school personnel. The survey was conducted in an anonymous manner, and a distinct study identification number was allocated to every student for data entry, management, and analysis. The personal information of the participants was kept confidential. Neither the teachers, parents, nor classmates knew your choices. Students could obtain their own measurement results from the school doctor using their identification codes. They would receive a free psychological assessment and medical help from the Health Education Department of the local Centers for Disease Control and Prevention within 24 hours if they had any mental health concerns, as compensation for their participation.

## Results

### Characteristics of the Study Population

The characteristics of the study population according to gender are shown in Table 1. Among the 26,394 adolescents, the mean age was 15.9 (SD 1.7) years, 48.6% (12,825/26,394) of them were girls, and 47.3% (12,492/26,394) were junior high school students. Girls were more likely to be locals (rate difference=2.4%; *χ*^2^_1_=21.6; *P*<.001), non–single child (rate difference=12.6%; *χ*^2^_1_=445.5; *P*<.001), live in dormitories (rate difference=1.7%; *χ*^2^_2_=9.6; *P*=.008), nonsmoking (rate difference=11.6%; *χ*^2^_1_=582.1; *P*<.001), drinking (rate difference=10.1%; *χ*^2^_1_=284.2; *P*<.001), wake lag of ≥1 hour per day (rate difference=11.5%; *χ*^2^_2_=562.0; *P*<.001), and bed lag of ≥1 hours per day (rate difference=6.7%; *χ*^2^_2_=45.8; *P*<.001). They had lower height (mean difference=10.23 cm; *t*_26,392_=106.88; *P*<.001), weight (mean difference=8.59 kg; *t*_26,392_=60.38; *P*<.001), and sleep duration (mean difference=0.26 hour; *t*_26,130_=14.20; *P*<.001), as well as higher PHQ-9 scores (mean difference=–0.59 points; *t*_24,671_=–23.10; *P*<.001) and GAD-7 scores (mean difference=–2.00 points; *t*_26,383_=–27.28; *P*<.001) than boys.

**Table 1:** Characteristics of adolescents by gender.

Characteristics		Combined (n=26,394)	Boys (n=13,569)	Girls (n=12,825)	*P* value	
**School types, n (%)**						<.001
	Junior high school	12,492 (47.3)	6510 (48)	5982 (46.6)		
	Academic high school	7226 (27.4)	3579 (26.4)	3647 (28.4)		
	Vocational high school	6676 (25.3)	3480 (25.7)	3196 (24.9)		
Age (years), mean (SD)		15.9 (1.7)	15.9 (1.7)	15.9 (1.8)	.07	
**Ethnicity, n (%)**						.96
	Han	25,422 (96.3)	13,070 (96.3)	12,352 (96.3)		
	Others	972 (3.7)	499 (3.7)	473 (3.7)		
**Registered permanent residence, n (%)**						<.001
	Local	20,598 (78)	10,433 (76.9)	10,165 (79.3)		
	Others	5796 (22)	3136 (23.1)	2660 (20.7)		
**Father’s education, n (%)**						.68
	Primary school or less	3097 (11.7)	1578 (11.6)	1519 (11.8)		
	Junior or senior high school	18,495 (70.1)	9541 (70.3)	8954 (69.8)		
	High school or above	4802 (18.2)	2450 (18.1)	2352 (18.3)		
**Mother’s education, n (%)**						.69
	Primary school or less	4492 (17)	2318 (17.1)	2174 (17)		
	Junior or senior high school	17,357 (65.8)	8892 (65.5)	8465 (66)		
	High school or above	4545 (17.2)	2359 (17.4)	2186 (17)		
**Parents’ marital status, n (%)**						<.001
	Married	22,992 (87.1)	11,923 (87.9)	11,069 (86.3)		
	Divorce	2549 (9.7)	1200 (8.8)	1349 (10.5)		
	Widowed	372 (1.4)	165 (1.2)	207 (1.6)		
	Separation	481 (1.8)	281 (2.1)	200 (1.6)		
Single child, n (%)		9971 (37.8)	5957 (43.9)	4014 (31.3)	<.001	
**Family economic status, n (%)**						<.001
	Poor	1593 (6)	901 (6.6)	692 (5.4)		
	Ordinary	22,574 (85.5)	11,393 (84)	11,181 (87.2)		
	Wealthy	2227 (8.4)	1275 (9.4)	952 (7.4)		
**Accommodation, n (%)**						.008
	Dormitories	11,940 (45.2)	6029 (44.4)	5911 (46.1)		
	Home	13,714 (52)	7134 (52.6)	6580 (51.3)		
	A rented room	740 (2.8)	406 (3)	334 (2.6)		
Height (cm), mean (SD)		166.5 (9.3)	171.5 (8.6)	161.3 (6.7)	<.001	
Weigh (kg), mean (SD)		56.8 (12.3)	61.0 (13.2)	52.4 (9.4)	<.001	
Smoking, n (%)		4984 (18.9)	3329 (24.5)	1655 (12.9)	<.001	
Drinking, n (%)		15,585 (59.1)	7339 (54.2)	8246 (64.3)	<.001	
Sleep duration^a^ (hour per day), mean (SD)		8.0 (1.5)	8.2 (1.5)	7.9 (1.5)	<.001	
PHQ-9^b^ score^c^ in points, mean (SD)		7.2 (2.0)	7.0 (2.0)	7.5 (2.0)	<.001	
GAD-7^d^ score^e^ in points, mean (SD)		8.4 (6.0)	7.4 (5.6)	9.4 (6.3)	<.001	
**Mobile phone placement habits^f^, n (%)**						<.001
	Beside pillow	11,601 (44.1)	6171 (45.7)	5430 (42.5)		
	Elsewhere	10,742 (40.9)	4964 (36.7)	5778 (45.2)		
	None	3947 (15)	2379 (17.6)	1568 (12.3)		
**Mobile phone use time on school days^g^, n (%)**						.005
	<1 hour per day	4762 (18.1)	2357 (17.4)	2405 (18.8)		
	≥1 hour per day	8403 (32)	4300 (31.8)	4103 (32.1)		
	None	13,125 (49.9)	6857 (50.7)	6268 (49.1)		
**Mobile phone average use time on weekends^g^, n (%)**						<.001
	<1 hour per day	2503 (9.5)	1376 (10.2)	1127 (8.8)		
	≥1 hour per day	21,254 (80.8)	10,590 (78.4)	10,664 (83.5)		
	None	2533 (9.6)	1548 (11.5)	985 (7.7)		
**Wake lag^a^, n (%)**						<.001
	<1 hour per day	6565 (25.1)	3536 (26.3)	3029 (23.9)		
	≥1 hour per day	16,147 (61.8)	7552 (56.2)	8595 (67.7)		
	None	3420 (13.1)	2348 (17.5)	1027 (8.4)		
**Bed lag^a^, n (%)**						<.001
	<1 hour per day	4331 (16.6)	2217 (16.5)	2114 (16.7)		
	≥1 hour per day	15,796 (60.5)	7906 (58.8)	7890 (62.2)		
	None	6005 (23)	3313 (24.7)	2692 (21.2)		

^a^Included 26,132 adolescents for analysis.

^b^PHQ-9: Patient Health Questionnaire 9-Item Scale.

^c^Included 24,673 adolescents for analysis.

^d^GAD-7: Generalized Anxiety Disorder 7-Item Scale.

^e^Included 26,385 adolescents for analysis.

^f^Included 26,290 adolescents for analysis.

^g^Included 26,290 adolescents for analysis, and excluded use of mobile phones for homework.

### Mobile Phone Use and the Prevalence of Suicidal Behaviors

The prevalence of suicidal behaviors according to mobile phone ownership and MPA is presented in Multimedia Appendix 3. There were statistically significant differences in the distribution of suicidal behaviors between adolescents with and without MPA (*χ*^2^_3_=1277.3; *P*<.001), but no significant differences were observed in relation to mobile phone ownership (*χ*^2^_3_=0.9; *P*=.83). Suicidal scores were higher in participants with MPA (*t*_26,392_=–34.12; *P*<.001) and in participants without mobile phone ownership (*t*_26,392_=2.38; *P*=.02).

### Mobile Phone Use With Associated Factors

Mobile phone ownership and MPA were associated with several characteristics in logistic regression models (Multimedia Appendix 4). Gender, school types, parents’ marital status, family economic status, accommodation, height, smoking, and drinking were independently associated with MPA, with an OR of 1.942 (95% CI 1.779-2.120) in girls, 1.389 (95% CI 1.213-1.591) in academic high school and 0.835 (95% CI 0.727-0.958) in vocational high school, 1.265 (95% CI 1.136-1.409) in divorce and 1.389 (95% CI 1.108-1.765) in separation, 0.572 (95% CI 0.506-0.647) in ordinary family economic status and 0.703 (95% CI 0.595-0.831) in wealthy, 0.876 (95% CI 0.814-0.944) in living at home and 0.794 (95% CI 0.641-0.983) in a rented room, 1.007 (95% CI 1.002-1.012) in height, 2.116 (95% CI 1.950-2.296) in smoking, and 1.732 (95% CI 1.610-1.863) in drinking. The associations with mobile phone ownership were similar to the above characteristics, but not with age, registered permanent residence, parents’ education, single child, height, or weight.

### Associations of Mobile Phone Use With Suicidal Behaviors

In Table 2, logistic regression analysis shows significant associations between mobile phone use and suicidal behaviors. In the crude model, compared with those without ownership, mobile phone ownership had an average decrease of 0.04 points in suicide score (β coefficient=–0.036, 95% CI –0.066 to –0.006), but it was not related to suicidal behaviors. Compared to the normal, MPA was over 2.7 times more likely to report suicide ideation (OR 2.717, 95% CI 2.491-2.964) and was over 5.3 times more likely to report suicide attempts (OR 5.267, 95% CI 4.420-6.276). Compared with suicide ideation, MPA was significantly associated with suicide plans (OR 1.553, 95% CI 1.341-1.752). Further, compared with suicide plans, MPA was significantly associated with suicide attempts (OR 1.264, 95% CI 1.033-1.548). MPA was positively associated with suicidal scores (β coefficient=0.489, 95% CI 0.461-0.517).

**Table 2:** Association of mobile phone ownership, mobile phone addiction (MPA), and suicidal behaviors in adolescents.

Outcomes			Mobile phone ownership			MPA		
			Crude model	Adjusted model^a^		Crude model		Adjusted model^a^
**Suicide behaviors, OR^b^ (95% CI)^c^**								
	Ideation vs normal	0.967 (0.873 to 1.071)		1.028 (0.923 to 1.146)	2.717 (2.491 to 2.964)		2.449 (2.236 to 2.683)	
	Plans vs ideation	1.078 (0.905 to 1.286)		1.078 (0.897 to 1.296)	1.533 (1.341 to 1.752)		1.453 (1.265 to 1.669)	
	Attempts vs plans	0.992 (0.751 to 1.311)		1.059 (0.791 to 1.419)	1.264 (1.033 to 1.548)		1.105 (0.894 to 1.366)	
	Attempts vs normal	1.034 (0.814 to 1.314)		1.175 (0.910 to 1.516)	5.267 (4.420 to 6.276)		3.934 (3.263 to 4.743)	
Suicidal scores^d^, β coefficient (95% CI)			–0.036 (–0.066 to –0.006)	–0.015 (–0.045 to 0.015)		0.489 (0.461 to 0.517)		0.409 (0.381 to 0.437)

^a^Adjusted model was adjusted for gender, age, ethnicity, registered permanent residence, school types, father’s education, mother’s education, parents’ marital status, single child, family economic status, accommodation, height, weight, smoking, and drinking.

^b^OR: odds ratio.

^c^OR and 95% CI were calculated using multinomial logistic regression analysis.

^d^β coefficient and 95% CI were calculated using multivariate linear regression.

In the adjusted model, no significant differences were observed between mobile phone ownership and suicidal behaviors or suicidal scores. Compared with the normal, MPA was significantly related to increased odds of suicide ideation (OR 2.449, 95% CI 2.236-2.268) and suicide attempts (OR 3.449, 95% CI 3.263-4.743). Compared with suicide ideation, greater odds of suicide plans (OR 1.453, 95% CI 1.265-1.669) were shown in MPA. However, compared with suicide plans, no significant differences were observed between MPA and suicide attempts. The positive association between MPA and suicidal scores was retained (β coefficient=0.409, 95% CI 0.381-0.437).

**Figure 2:**
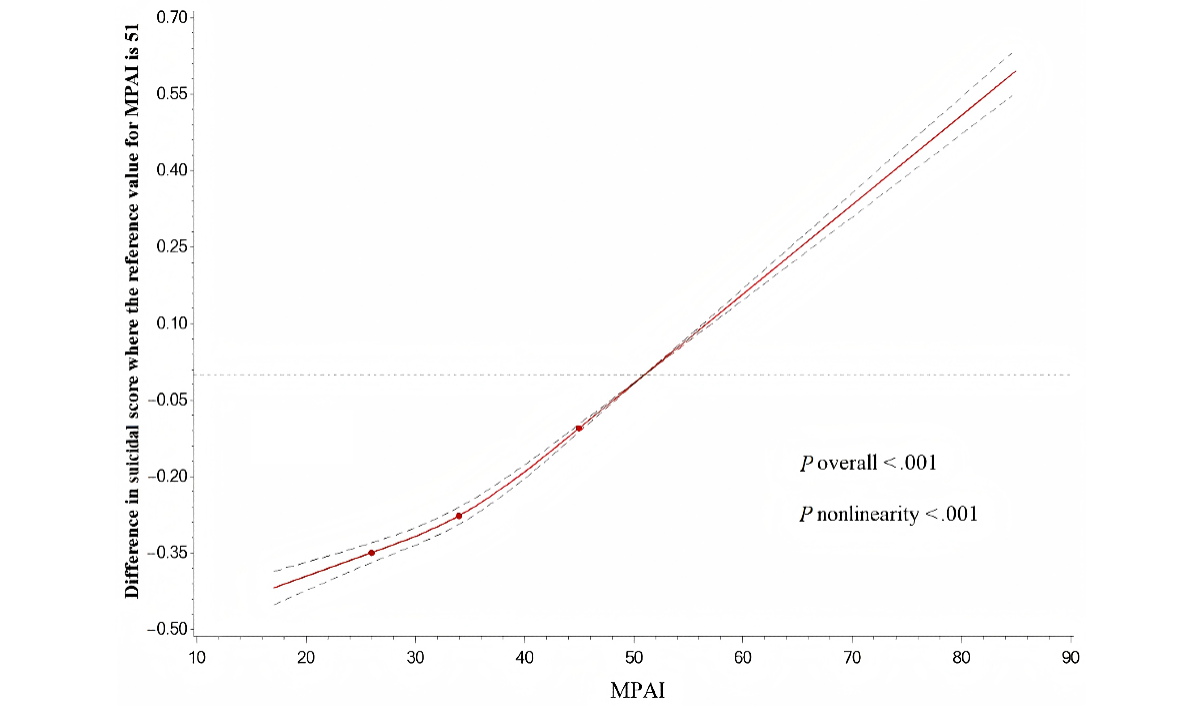
Adjusted dose-response association between MPAI and suicidal scores. MPAI were coded using a restricted cubic spline function with 3 knots located at the 25th, 50th, and 75th percentiles of the distribution of MPAI. y-axis represented the difference in suicidal scores between individuals with any value of MPAI with individuals with 51 of MPAI. Dashed lines were 95% CIs. Knots were represented by dots. MPAI: Mobile Phone Addiction Index.

As shown in Figure 2, linear regression with restricted cubic splines was conducted to model and visualize the association between MPAI and suicidal scores. As the MPAI increased, the suicide scores increased significantly. A nonlinear association between them was observed (nonlinearity *P*<.001). Multimedia Appendix 5 shows that the relationship between MPAI and suicide ideation (nonlinear *P*<.001) or suicide plans (nonlinear *P*<.001) was nonlinear, while MPAI and suicide attempts was linear (nonlinear *P*=.92).

**Figure 3:**
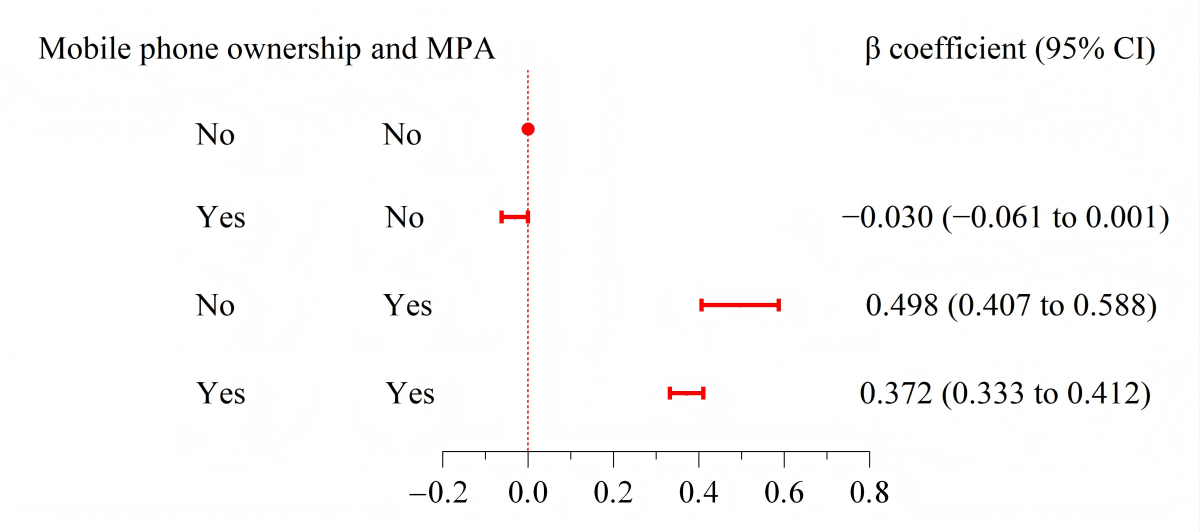
The association between interactions of mobile phone ownership and MPA and suicidal scores. The model was adjusted for the same variables as the adjusted model in Table 2. MPA: mobile phone addiction.

In Figure 3, we further examined the average suicidal score change based on categories of mobile phone ownership and MPA. Regardless of mobile phone ownership, the MPA group had higher suicidal scores compared with the participants without MPA. The MPA without mobile phone ownership had the highest increase in suicidal scores compared to those who had neither mobile phone ownership nor MPA. (β coefficient=0.498, 95% CI 0.407-0.588).

**Figure 4:**
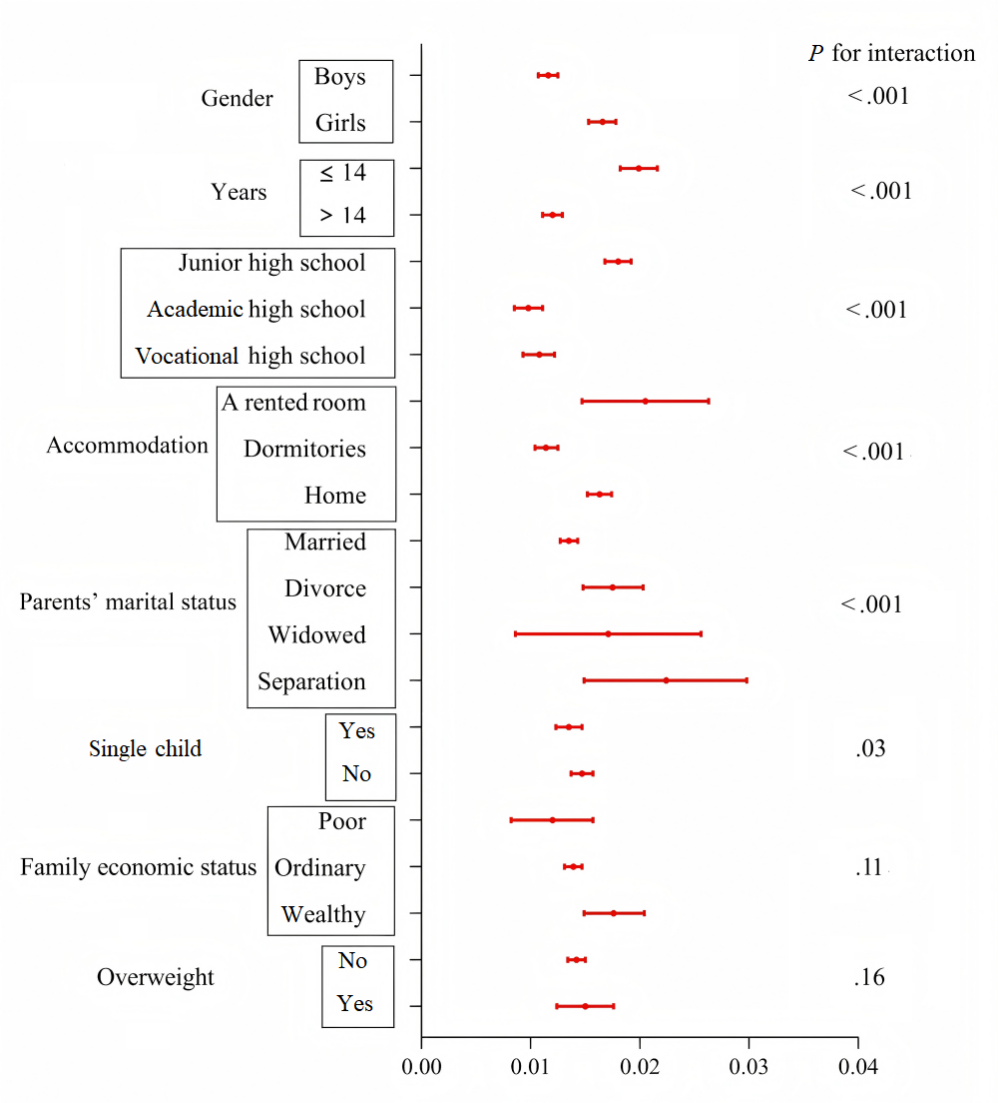
Association between Mobile Phone Addiction Index and suicidal scores across various subgroups using multivariate linear regression with coefficients β and 95% CIs. Analysis was adjusted for gender, age, ethnicity, registered permanent residence, school types, father’s education, mother’s education, parent’s marital status, single child, family economic status, accommodation, height, weight, smoking, and drinking other than variables for stratification.

Figure 4 presents the suicidal scores for the MPAI in various subgroups. The higher MPAI was consistently positively correlated with suicidal scores in all subgroups. This association was more robust in girls, ages ≤14 years, junior high school, living in a rented room, separated parents, and non–single child. There were no significant interactions of family economic status or overweight for the association between MPAI and suicidal scores.

### Sensitivity Analysis

Participants with mobile phone ownership seemed to prefer to keep mobile phones beside pillows. While the percentage of average use time per day of <1 hour on school days was low, the percentage of ≥5 hours on weekends was high. Most of the participants with MPA did not use their phones on school days, while the average use time per day of ≥5 hours on weekends was very high (Multimedia Appendix 6). In the sensitivity analysis, mobile phone placement habits and average use time per day on school days and weekends were entered in the adjusted model of Table 2. Mobile phone ownership was negatively correlated with suicidal scores (β coefficient=–0.087, 95% CI –0.127 to –0.048), while MPA was positively correlated with suicidal scores (β coefficient=0.396, 95% CI 0.367-0.425).

PSW regression models showed that mobile phone ownership was negatively associated with suicidal scores (β coefficient=–0.096, 95% CI –0.126 to –0.066). Compared with normal, mobile phone ownership was negatively associated with suicide ideation (OR 0.903, 95% CI 0.817-0.998). MPA was still positively related to suicide behaviors, as it was when MPAI ≥60 was used to define MPA (Multimedia Appendices 7 and 8).

An *E*-value was calculated to assess the potential impact of unaccounted confounders. The *E*-value for the standardized mean difference was 2.42 and 2.32 for the lower CI. These confounding factors would need to have a risk ratio of at least 2.32 to account for the association between MPA and suicidal scores (Multimedia Appendix 9).

### Mediation Analysis

**Figure 5:**
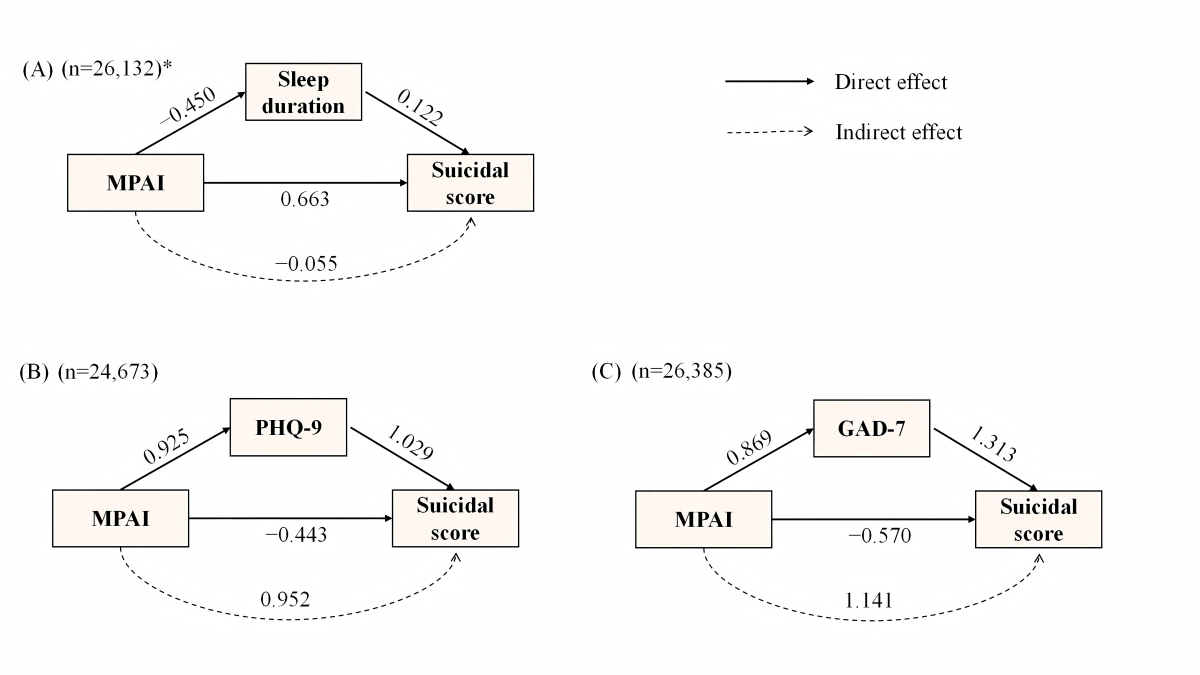
(A) Mediation effect of sleep duration and (B) PHQ-9 scores and (C) GAD-7 scores on the MPAI-suicidal scores associations. Standardized coefficients β were presented. GAD-7: Generalized Anxiety Disorder 7-Item Scale; MPAI: Mobile Phone Addiction Index; PHQ-9: Patient Health Questionnaire 9-Item Scale. *The model was further adjusted for wake and bed lag.

Significant statistical relationships were found between sleep duration, depression and anxiety, MPAI, and suicidal scores (Multimedia Appendix 10; all *P*<.001). As Figure 5A-C shows, after controlling for covariates, there were standardized direct effects of MPAI (β coefficient=0.663, 95% CI 0.645-0.671; *P*<.001) and sleep duration (β coefficient=–0.450, 95% CI –0.459 to –0.441; *P*<.001) on suicidal scores. The standardized total effect of MPAI on suicidal scores was 0.608 (95% CI 0.601-0.615; *P*<.001), and the standardized indirect effect was –0.055 (95% CI –0.060 to –0.050; *P*<.001). The mediation ratio was 9%. Notably, depression (standardized indirect effect: β coefficient=0.952, 95% CI 0.927-0.977; *P*<.001) and anxiety (standardized indirect effect: β coefficient=1.141, 95% CI 1.130-1.152; *P*<.001) partially mediated the association between MPAI and suicidal scores. The mediation ratio of the PHQ-9 score was 187.4%, and the mediation ratio of the GAD-7 score was 199.8%.

## Discussion

### Principal Findings

In this school-based cross-sectional study, a large sample of 26,394 Chinese adolescents was used to evaluate the association between mobile phone use and suicidal behaviors. MPA, rather than mobile phone ownership, was positively associated with both suicidal behaviors and suicidal scores. MPA without mobile phone ownership was positively associated with suicidal scores. We found that the odds of suicidal behaviors and their scores significantly increased with MPAI. Notably, MPAI also had an indirect effect on suicide scores, with a mediating role of low sleep duration, high PHQ-9 score, and high GAD-7 score. These findings advance our understanding of the development of suicidal behaviors in adolescents in the context of the seeming inability to put their mobile phones down.

### Mobile Phone Use and Suicide Behaviors

Previous findings on the relationship between MPA and suicidal behaviors were inconsistent. In Chinese adolescents with a mean age of 15.0 (SD 1.6) years, Cheng et al [16] found that MPA was significantly associated with suicide ideation and plans, but not with suicide attempts, whereas Chen et al [17] found that MPA was associated with ideation and attempts, but not with its plans. MPA was associated with suicidal ideation and plans in American and Korean adolescents [13,19,45]. In this study, MPA was independently associated with suicide ideation and attempts, and MPA had higher odds of suicide plans compared with ideation. In contrast to our study design, Noel et al [19] used the Smartphone Addiction Scale-Short Version consisting of 10 items, Shinetsetseg et al [13] and Lee and Lee [45] used the Korean version of the smartphone overdependence scale, and Chen et al [17] used a self-designed questionnaire, which included only 2 questions regarding mobile phone use duration to assess MPA, rather than using the MPAI scale. Additionally, similar to this study, these studies were conducted during the COVID-19 pandemic—a period that prompted many countries worldwide to implement quarantine protocols (eg, more than 220 million Chinese students began online classes at home via smartphones or tablets, which could result in an emerging prevalence of MPA [46]). Besides, a nonlinear relationship between MPAI and suicidal ideation and plans was found, respectively, whereas MPAI and attempts were linear. A cross-sectional study of 18,723 college students consistently showed a nonlinear dose-response association between MPA tendency scale score—a scale to measure MPA for college students and suicide ideation, whereas a linear relationship with attempts [47]. Given the differences in these findings, further longitudinal investigations or intervention studies on the relationship between MPA and suicidal behaviors in nationally representative samples using standardized measurement tools are warranted. Notably, girls had greater odds of suicide ideation, plans, and attempts than boys [16], which was consistent with ours that the relationship between MPAI and suicidal scores was stronger in girls than boys. It was reported that girls had more communication using social networking service desires than boys [48]. When interpreting the significant association between MPA and suicide behaviors, it was necessary to consider the differences in the distribution of the study population characteristics, such as family education concepts and school management policies, especially the impact of changes in lifestyle during the COVID-19 pandemic [49].

### Mediating Mechanisms

In the internet era, MPA was similar to internet addiction, both of which were technology addictions with similar characteristics and formation mechanisms [50], usually accompanied by withdrawal symptoms, tolerance, loss of control, and adverse reactions [51]. A meta-analysis showed that there was an association between excessive internet use and the risk of suicide in adolescents [52]. The internet or smartphone provided and created many opportunities related to suicidal issues, such as online movies and chat rooms where experiences of suicide were discussed and shared. Adolescents could learn about suicidal behaviors and actual methods of suicide [53]. The World Health Organization also suggested that measures should be taken to restrict or regulate the dissemination and reporting of online content related to suicide [54]. In addition, a cohort study of 4285 US adolescents found that addictive screen use (social media, mobile phones, and video games), rather than total screen time, was associated with suicidal behavior [55]. Interventions targeting adolescent screen addiction may prioritize functionally restricted devices (eg, smartphones with social or gaming applications removed) in future trials.

Our findings supported the mediating role of depression in the association between MPA and suicidal behaviors [17,49]. Adolescents with addictive behaviors on the internet showed high levels of loneliness, impulsivity, irritation, anger, and other adverse reactions related to depression [56]. MPA could lead to work or school performance impairments [57], which may cause severe criticism from teachers and family members. The generation of negative emotions may lead to more compulsive use of mobile phones to escape these real-world troubles, which may increase the risk of cyberbullying victimization [9,58] and induce subsequent suicidality. Similarly, anxiety mediated the relationship between MPA and suicide behaviors. A study of 18,980 participants aged ≥15 years from 5 countries found that social anxiety significantly increased the risk of major depression [59]. MPA was significantly associated with higher levels of anxiety and suicide ideation in Korean adolescents [45]. Notably, the standardized direct effect of MPA on suicidal scores was negative, whereas its standardized indirect effect was positive. This suppression effect [60] indicates that the influence of MPA may be bidirectional. Hu et al [61] demonstrated that online social support could moderate the relationships between MPA and depression, as well as between MPA and suicidal ideation. Adolescents could obtain emotional and social support resources via mobile phones, which influenced their coping and appraisal, thereby protecting them from mental health problems [62]. Notably, resting-state functional magnetic resonance imaging revealed that problematic smartphone users exhibited enhanced functional connectivity between the amygdala and dorsolateral prefrontal cortex while showing reduced connectivity between the right subgenual anterior cingulate cortex and lingual gyrus. These findings imply an imbalance in emotional control and impairments in their ability to perceive or regulate in individuals [63].

In line with present studies, MPA may affect sleep quality and increase the prevalence of suicidal behaviors [16,49]. High levels of MPA severity were significantly associated with poor sleep quality [24]. Individuals with MPA tend to spend more time using their phones at night, which may increase the risk of insomnia, excessive daytime sleepiness, short total sleep time, and circadian rhythm disruption [64]. The conflict between late sleep and early wake-up on school days may also prevent students from obtaining sufficient sleep. Therefore, we adjusted wake and bed lag (schooldays vs weekends) in the mediation model to account for the potential confounding effects caused by circadian misalignment or “social jetlag” [65]. In addition, screen lights from electronic devices affect serum melatonin and cerebral blood flow, which leads to poor sleep quality [66,67]. Individuals with poor sleep quality have impaired frontal lobe and executive function, causing emotional regulation disorders and impulsive behaviors [68]. Moreover, their hypothalamus or hypothalamic-pituitary-adrenal axis was dysregulated, which was associated with suicidal behaviors [69]. Longitudinal studies to collect relevant biological and neuroscience data would be helpful to further dig into the mechanisms of MPA suicide and to provide directions for clinical trials targeting therapeutic targets.

### Mobile Phone Ownership and Suicidal Behaviors

Whether and when kids should get their first mobile phone? The question of the harmful or beneficial of owning a mobile for kids has frequently plagued many parents, educators, and clinicians. Prior studies in Chinese adolescents primarily focused on MPA, which may leave a critical gap in mobile phone ownership as a risk factor in suicidal behaviors. To our knowledge, empirical studies examining the association between mobile phone ownership and suicidal behaviors in Chinese adolescents remain limited. Findings suggested an absence of a statistical link between mobile phone ownership and suicide behaviors but a significant negative link with suicide score in sensitive analysis. A convenience sample of urban youths aged 14-24 years living on the streets or in the slums indicated that mobile phone ownership was not related to suicide attempt [70]. However, adolescent with mobile phone ownership was related to the risk of unhealthy behaviors such as substance use, with greater odds of using nicotine or tobacco and cannabis [70,71]. Mobile phone ownership may not be related to the occurrence of adjustment measures of depression and sleep [72] but was positively related to psychological discomfort, problematic use, and imitation of dangerous behaviors [73]. The association of mobile phone ownership with a variety of negative psychosocial and unhealthy behaviors raises the possibility of subsequent suicidality. However, the uncertainty of the above associations needs to be improved by representative prospective studies in the future.

Instead, researchers have focused more on the appropriate age for kids to get their own smartphones [74]. Thiagarajan et al [75] found that receiving a smartphone before 13 years of age was associated with poorer mental health outcomes in young adulthood. This study population with a mean age of 16 years revealed that no association was observed between mobile phone ownership and suicidal behaviors. However, in subgroup analysis, the association between MPAI and suicide score in the group aged ≤14 years was stronger than that in the group aged >14 years. We hypothesize that the earlier the age of initial mobile phone exposure, the more significant the adverse effects of MPA will be under the context of the developmental vulnerability window. In contrast, as age increases, the maturation of mental functioning partially attenuates the association between MPA and suicide behaviors [76-78]. We found a weak inverse association between mobile phone ownership and suicidal scores in sensitivity analysis, whereas MPA without mobile phone ownership was associated with the highest suicide scores. The change from a categorical variable (suicidal behaviors) to a continuous variable (suicidal scores) may reduce statistical power. More importantly, Martín-Cárdaba et al [73] found that when parents actively discuss the potential dangers and distortions of social media with their children, it could prevent them from experiencing psychological trauma. King et al [23] found that people tend to experience “separation anxiety” when without a mobile phone. The emotional dependence arising from the fear of being unable to communicate or seek help due to the absence of a mobile phone has led to “nomophobia” [23]. Therefore, providing adolescents with shared or restricted access to mobile phones under parental supervision holds significance in the prevention of suicidal behaviors. With the effect of these factors in mind, mobile phone ownership may be “later is better.”

### Strengths and Limitations

The strengths of our study included a large sample in China with a rigorous multistage cluster sampling method, in which potential confounding factors were collected as far as possible. More importantly, we examined the mediating roles of physiological (sleep duration) and psychological (depression and anxiety) factors, which added further and reliable evidence for the relationship between MPA and suicidal behaviors. Besides, the visual association of restricted cubic splines and the sensitivity analysis of PSW made the results more stable and reliable.

Limitations need to be kept in mind when interpreting the results. First, this study was a cross-sectional study and was not randomized. The ability to clarify the cause-effect of mobile phone use and suicidal behaviors in adolescents was limited. Therefore, it was necessary to dynamically monitor the changes in mobile phone use and suicidality in a prospective cohort study, especially the impact of changes in lifestyle and psychology in adolescents before and after the COVID-19 pandemic. In addition, the purposes of mobile phone use of the participants with MPA were not collected in this study. Future studies should explicitly distinguish between use scenarios (eg, recreational vs learning-oriented use) and help identify more targeted intervention points (eg, clarifying that excessive or problematic social media use, rather than educational mobile use, correlates with higher suicide risk). Finally, suicidal behaviors were assessed using a structured questionnaire. The questionnaire only identified those who have engaged in suicidal behaviors at least once in the past 12 months but cannot assess the frequency and severity of such behaviors. Besides, similar to the binary suicidal behavior items in previous studies [16,17], we also failed to capture details such as the frequency of suicidal ideation or the severity of suicidal behaviors, which may have restricted the depth of our analysis of suicidal experiences. While we converted suicidal behavior into a continuous variable to capture the gradation of its severity, implementing more nuanced evaluations for Chinese adolescents warranted careful consideration [79]. Meanwhile, the data were collected using self-reported measures, which inevitably introduced recall bias. Immediate measurement of suicidality, such as suicide-related behaviors in the past 30 days or hospital admissions linked to suicide attempts, may help reduce recall biases.

### Conclusions

MPA, rather than mobile phone ownership, was independently related to increased odds of suicidal ideation, plans, and attempts. MPA without mobile phone ownership was related to higher odds of suicidality than the absence of both. These findings highlighted the need for parents and educators to acknowledge the issues related to smartphone overuse or addiction, and adolescents with MPA but no mobile phone ownership represented a high-risk subgroup. Furthermore, sleep duration, depression, and anxiety partially mediated the link between MPA and suicidality. A meta-analysis of randomized controlled trials demonstrated that smartphone app-based e-therapies effectively alleviate depression and anxiety [80]. Intervention programs could target adolescents with higher MPAI, conduct screening for depression and anxiety, and implement sleep hygiene interventions to mitigate the effects of MPA.

Given our findings, the potential targets of intervention (MPA behavior, not device ownership) as well as the multipathway mechanisms have been indicated. In practice, we propose the following recommendations: (1) schools should incorporate screening for problematic mobile phone use into mental health screenings, with a focus on high-risk subgroups, and provide emotional guidance alongside the implementation of sleep hygiene programs for adolescents; (2) while prioritizing children’s privacy, parents shift from “ownership restriction” to “behavior management,” using functionally restricted phones with emotional support; and (3) policymakers should emphasize incorporating MPA into mental health and develop mental health guidelines for smartphone use among adolescents. Such research could inform evidence-based policies and practices designed to promote digital well-being among adolescents, ultimately contributing to their psychological resilience in the face of challenges related to suicidality.

## Appendix

Multimedia Appendix 1 The definition of the classification of suicidal behaviors.

Multimedia Appendix 2 The suicidal behavior questionnaire and its scoring.

Multimedia Appendix 3 Bar graphs showing the prevalence of suicidal behaviors according to mobile phone ownership and mobile phone addiction. (A and B) Suicidal behaviors were presented as a suicide-related behaviors questionnaire scores. (C and D) Suicide behaviors were classified into ideation, plans, and attempts.

Multimedia Appendix 4 Associated factors with mobile phone ownership or mobile phone addiction form multivariable logistic regression models.

Multimedia Appendix 5 Adjusted dose-response associations between MPAI and suicidal behaviors. MPAI: Mobile Phone Addiction Index; OR: odds ratio.

Multimedia Appendix 6 Bar graphs showing the prevalence of mobile phone placement habits and its school days and weekends use times according to mobile phone ownership and mobile phone addiction.

Multimedia Appendix 7 Sensitivity analysis in the associations of mobile phone ownership, mobile phone addiction, and suicidal behaviors in adolescents.

Multimedia Appendix 8 Sensitivity analysis in the associations of mobile phone addiction and suicidal behaviors in adolescents.

Multimedia Appendix 9 Curves of the sensitivity analysis for unobserved confounders with E-value. Curve depicting the range of joint relationships (MPA-confounders and confounders-suicidal scores) that may explain away the estimated effect and its CI for the multivariate linear regression to predict suicidal scores. A larger E-value indicated greater robustness of the research conclusion and made it more difficult for confounding factors to explain or overturn the results. MPA: Mobile Phone Addiction.

Multimedia Appendix 10 The association of Mobile Phone Addiction Index, suicidal scores, and mediating factors with β coefficient and CI.

## Abbreviations

GAD-7: Generalized Anxiety Disorder 7-Item Scale
MPA: mobile phone addiction
MPAI: Mobile Phone Addiction Index
OR: odds ratio
PHQ-9: Patient Health Questionnaire 9-Item Scale
PSW: propensity score weighting

## Data Availability

The datasets used during this study are available from the corresponding author on reasonable request.
