# Supplementary material for: Mobile Phone Addiction and Suicidal Behaviors in Adolescents: School-Based Cross-Sectional Study in Zhejiang Province, China
Source: J Med Internet Res. 2025 Nov 24;27:e80410. doi: 10.2196/80410 (PMC12686853; doi:10.2196/80410)
Supplement: Multimedia Appendix 1 [file jmir_v27i1e80410_app1.docx]

|  | | | |
| --- | --- | --- | --- |
| Suicidal behaviors | Suicide-related behaviors questionnaires | | |
|  | Serious thoughts of suicide have occurred during the past year | Had made a specific suicide plan during the past year | Had tried to suicide during the past year |
| Normal | No | - | - |
| Suicide ideation | Yes | No | - |
| Suicide plans | Yes | Yes | No |
| Suicide attempts | Yes | Yes | Only one/ Twice or more |
