# Supplementary material for: Mobile Phone Addiction and Suicidal Behaviors in Adolescents: School-Based Cross-Sectional Study in Zhejiang Province, China
Source: J Med Internet Res. 2025 Nov 24;27:e80410. doi: 10.2196/80410 (PMC12686853; doi:10.2196/80410)
Supplement: Multimedia Appendix 2 [file jmir_v27i1e80410_app2.docx]

|  | |
| --- | --- |
| Item1: Have you seriously considered suicide in the last 12 months? | |
| Response | Points |
| Never | 1 |
| Yes | 2 |
| Item2: Have you made a specific plan to commit suicide in the last 12 months? | |
| Response | Points |
| Never | 1 |
| Yes | 2 |
| Item3: Have you attempted suicide in the last 12 months? | |
| Response | Points |
| Never | 1 |
| 1 time | 2 |
| ≥ 2 times | 3 |
| Item4: If you have committed suicide, have you been hospitalized for injuries in the past 12 months? | |
| Response | Points |
| Never | 1 |
| Yes, but I didn't go to the hospital | 2 |
| Yes, I went to the hospital | 3 |
| Total points | 4 to 10 |
