# Supplementary figures and images for: Mobile Phone Addiction and Suicidal Behaviors in Adolescents: School-Based Cross-Sectional Study in Zhejiang Province, China
Source: J Med Internet Res. 2025 Nov 24;27:e80410. doi: 10.2196/80410 (PMC12686853; doi:10.2196/80410)

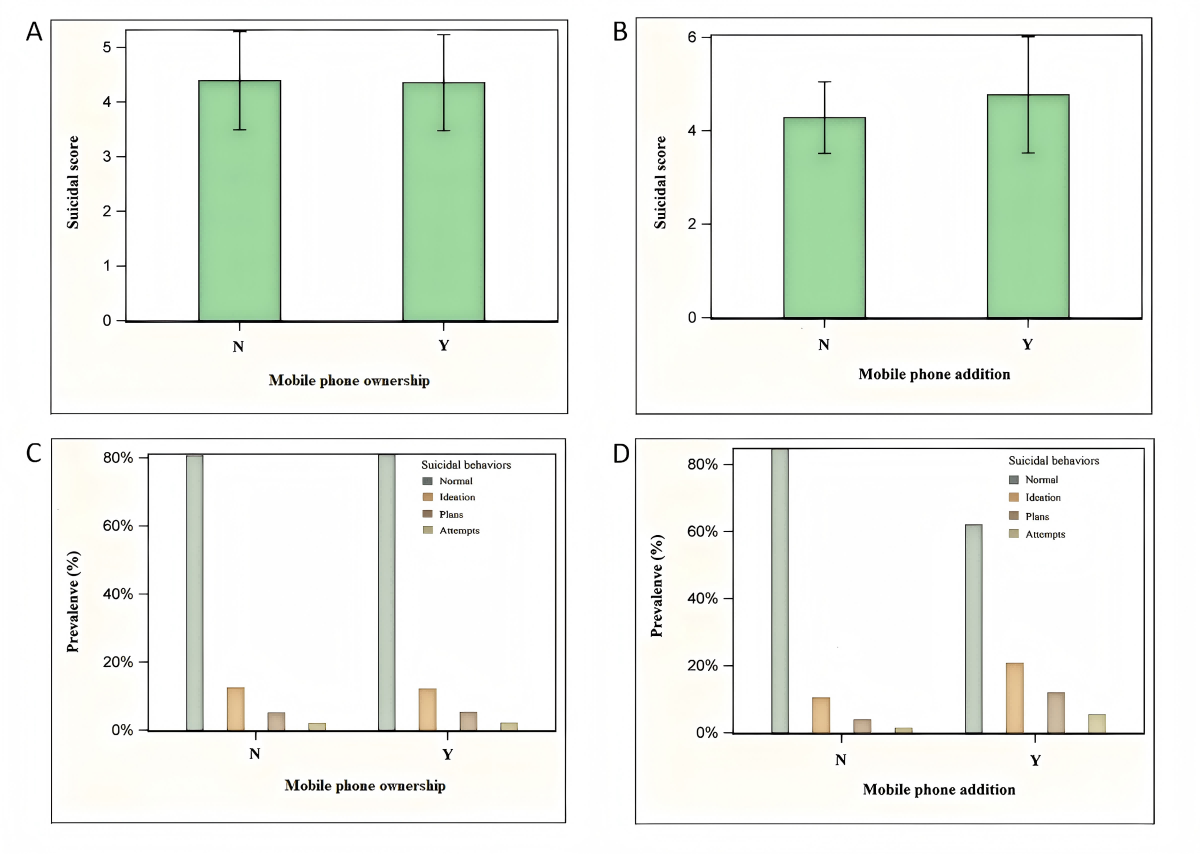

Supplement: Multimedia Appendix 3 [file jmir_v27i1e80410_app3.png]

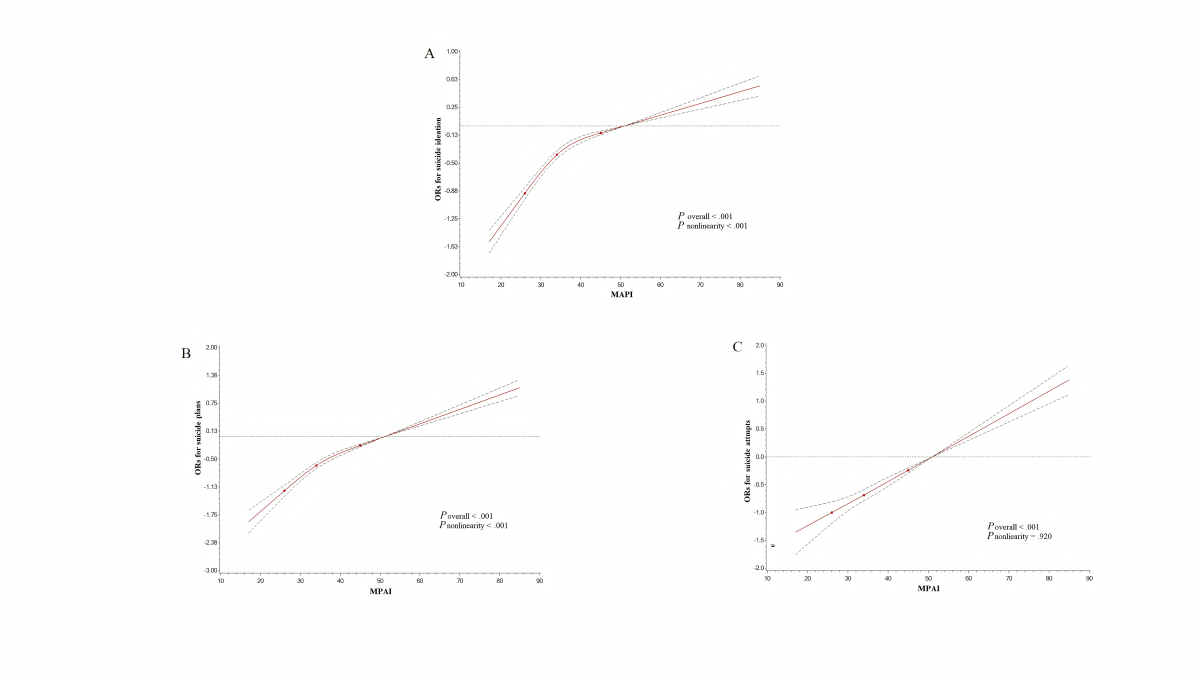

Supplement: Multimedia Appendix 5 [file jmir_v27i1e80410_app5.png]

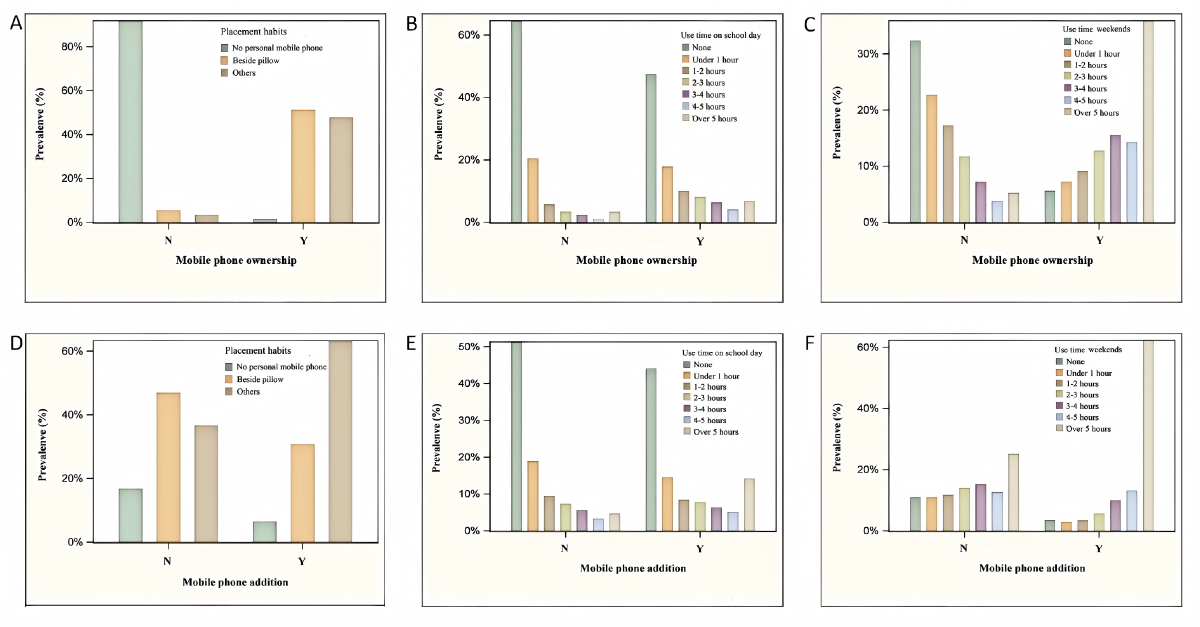

Supplement: Multimedia Appendix 6 [file jmir_v27i1e80410_app6.png]

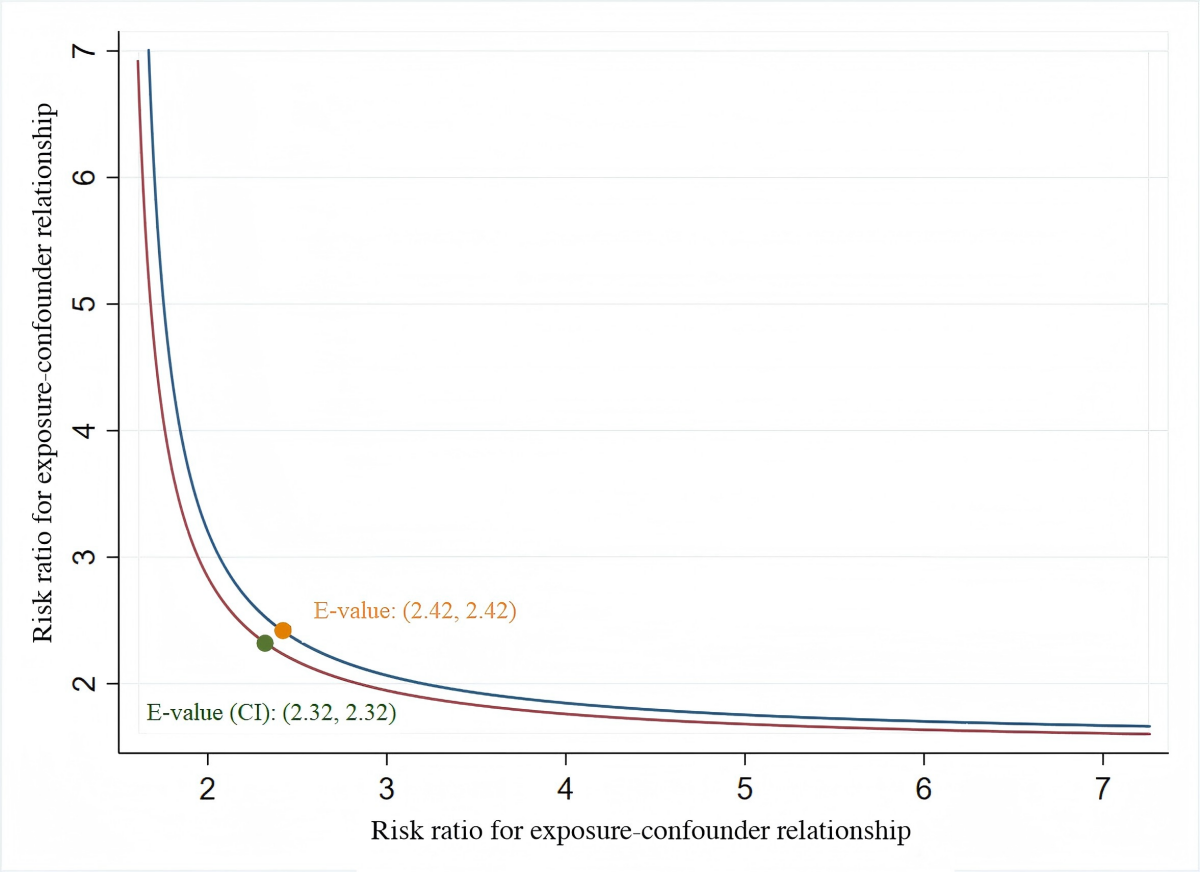

Supplement: Multimedia Appendix 9 [file jmir_v27i1e80410_app9.png]
