# Supplementary material for: Mobile Phone Addiction and Suicidal Behaviors in Adolescents: School-Based Cross-Sectional Study in Zhejiang Province, China
Source: J Med Internet Res. 2025 Nov 24;27:e80410. doi: 10.2196/80410 (PMC12686853; doi:10.2196/80410)
Supplement: Multimedia Appendix 4 [file jmir_v27i1e80410_app4.docx]

|  | | | | | | |  |
| --- | --- | --- | --- | --- | --- | --- | --- |
| Variables | | Mobile phone ownership | |  | MPA | |  |
|  |  | OR (95% CI) | *P* value | OR (95% CI) | | *P* value |  |
| Gender | |  | <.001 |  |  | <.001 |  |
|  | Boys | Ref. |  |  | Ref. |  |  |
|  | Girls | 1.626 (1.497 to 1.765) |  |  | 1.942 (1.779 to 2.120) |  |  |
| School types | |  | <.001 |  |  | <.001 |  |
|  | Junior high school | Ref. |  |  | Ref. |  |  |
|  | Academic high school | 1.667 (1.437 to 1.933) |  |  | 1.389 (1.213 to 1.591) |  |  |
|  | Vocational high school | 5.678 (4.693 to 6.871) |  |  | 0.835 (0.727 to 0.958) |  |  |
| Ages, years | | 1.203 (1.156 to 1.252) | <.001 |  | 1.012 (0.976 to 1.050) | .52 |  |
| Ethnicity | |  | .028 |  |  | .26 |  |
|  | Han | 0.81 (0.671 to 0.977) |  |  | 0.905 (0.76 to 1.078) |  |  |
|  | Others | Ref. |  |  | Ref. |  |  |
| Registered permanent residence | |  | .002 |  |  | .39 |  |
|  | local | Ref. |  |  | Ref. |  |  |
|  | Others | 0.873 (0.800 to 0.952) |  |  | 0.963 (0.885 to 1.048) |  |  |
| Father’s education | |  | <.001 |  |  | .06 |  |
|  | Primary school or less | Ref. |  |  | Ref. |  |  |
|  | Junior or senior high school | 0.861 (0.756 to 0.979) |  |  | 0.928 (0.835 to 1.031) |  |  |
|  | High school or above | 0.712 (0.606 to 0.837) |  |  | 0.834 (0.718 to 0.968) |  |  |
| Mother’s education | |  | .002 |  |  | .400 |  |
|  | Primary school or less | Ref. |  |  | Ref. |  |  |
|  | Junior or senior high school | 0.974 (0.873 to 1.087) |  |  | 0.961 (0.875 to 1.055) |  |  |
|  | High school or above | 0.801 (0.691 to 0.929) |  |  | 0.906 (0.785 to 1.045) |  |  |
| Parents’ marital status | |  | <.001 |  |  | <.001 |  |
|  | Married | Ref. |  |  | Ref. |  | |
|  | Divorce | 1.802 (1.557 to 2.086) |  |  | 1.265 (1.136 to 1.409) |  | |
|  | Widowed | 1.469 (1.026 to 2.103) |  |  | 1.184 (0.913 to 1.536) |  |  |
|  | Separation | 1.744 (1.307 to 2.328) |  |  | 1.398 (1.108 to 1.765) |  |  |
| Single child | | 1.146 (1.060 to 1.239) | .001 |  | 0.946 (0.878 to 1.019) | .14 |  |
| Family economic status | |  | <.001 |  |  | <.001 |  |
|  | Poor | Ref. |  |  | Ref. |  |  |
|  | Ordinary | 1.610 (1.382 to 1.875) |  |  | 0.572 (0.506 to 0.647) |  |  |
|  | Wealthy | 1.842 (1.523 to 2.228) |  |  | 0.703 (0.595 to 0.831) |  |  |
| Accommodation | |  | <.001 |  |  | .001 |  |
|  | Dormitories | Ref. |  |  | Ref. |  |  |
|  | Home | 0.710 (0.654 to 0.772) |  |  | 0.876 (0.814 to 0.944) |  |  |
|  | A rented room | 0.549 (0.453 to 0.666) |  |  | 0.794 (0.641 to 0.983) |  |  |
| Height, cm | | 1.005 (1.000 to 1.010) | .05 |  | 1.007 (1.002 to 1.012) | .010 |  |
| Weight, kg | | 1.004 (1.000 to 1.007) | .032 |  | 0.998 (0.994 to 1.001) | .19 |  |
| Smoking | | 1.157 (1.036 to 1.292) | .009 |  | 2.116 (1.950 to 2.296) | <.001 |  |
| Drinking | | 1.290 (1.188 to 1.401) | <.001 |  | 1.732 (1.610 to 1.863) | <.001 |  |
| ORs (95% CI) and P values estimated with Logistic regression model adjusted for all other characteristics. OR, odd ratio; CI, confidence interval; MPA, mobile phone addiction. | | | | | | |  |
