# Supplementary material for: Mobile Phone Addiction and Suicidal Behaviors in Adolescents: School-Based Cross-Sectional Study in Zhejiang Province, China
Source: J Med Internet Res. 2025 Nov 24;27:e80410. doi: 10.2196/80410 (PMC12686853; doi:10.2196/80410)
Supplement: Multimedia Appendix 7 [file jmir_v27i1e80410_app7.docx]

|  | | | | | | |
| --- | --- | --- | --- | --- | --- | --- |
| Outcomes | | Mobile phone ownership | |  | MPA | |
|  |  | Model 1 | Model 2 |  | Model 1 | Model 2 |
| suicidal behaviors^a^ | | | | | | |
|  | Ideation vs. Normal^c^ | 1.073 (0.839 to 1.371) | 0.903 (0.817 to 0.998) |  | 2.239 (2.033 to 2.466) | 2.411 (2.213 to 2.628) |
|  | Plans vs. Ideation ^c^ | 0.815 (0.547 to 1.215) | 1.036 (0.873 to 1.228) |  | 1.414 (1.219 to 1.641) | 1.709 (1.503 to 1.943) |
|  | Attempts vs. Plans ^c^ | 1.251 (0.679 to 2.307) | 0.946 (0.726 to 1.233) |  | 1.097 (0.873 to 1.379) | 0.907 (0.742 to 1.109) |
|  | Attempts vs. Normal^c^ | 1.095 (0.642 to 1.867) | 0.885 (0.706 to 1.109) |  | 3.473 (2.839 to 4.248) | 3.738 (3.131 to 4.463) |
| Suicidal scores^b^ | | −0.087 (−0.127 to −0.048) | −0.096 (−0.126 to −0.066) |  | 0.396 (0.367 to 0.425) | 0.432 (0.404 to 0.460) |
| Model 1 was adjusted for the same variables as adjusted model in Table 2 and for mobile phone placement habits, average usage time per day on schooldays and weekends (n=26,290). Model 2 was was adjusted for the same variables as adjusted model in Table 2 using propensity score weighting regression. a: odds ratio and confidence interval were calculated using multinomial Logistic regression analysis. b: coefficients β and confidence interval were calculated using multivariate linear regression. c: Reference group. MPA, mobile phone addiction. | | | | | | |
