# Supplementary material for: Mobile Phone Addiction and Suicidal Behaviors in Adolescents: School-Based Cross-Sectional Study in Zhejiang Province, China
Source: J Med Internet Res. 2025 Nov 24;27:e80410. doi: 10.2196/80410 (PMC12686853; doi:10.2196/80410)
Supplement: Multimedia Appendix 8 [file jmir_v27i1e80410_app8.docx]

|  | | |
| --- | --- | --- |
| Outcomes | | MPA^d^ |
| Suicidal behaviors^a^ | | |
|  | Ideation vs. Normal^c^ | 4.967 (4.018 to 6.140) |
|  | Plans vs. Ideation ^c^ | 1.731 (1.463 to 2.047) |
|  | Attempts vs. Plans ^c^ | 1.777 (0.929 to 1.492) |
|  | Attempts vs. Normal^c^ | 2.438 (2.154 to 2.758) |
| Suicidal scores^b^ | | 0.549 (0.510 to 0.589) |
| Model was adjusted for the same variables as adjusted model in Table 2. a: odds ratio and confidence interval were calculated using multinomial logistic regression analysis. b: β coefficients and confidence interval were calculated using multivariate linear regression. c: reference group. d: MPAI ≥ 60. MPA, mobile phone addiction. | | |
