# Supplementary material for: Mobile Phone Addiction and Suicidal Behaviors in Adolescents: School-Based Cross-Sectional Study in Zhejiang Province, China
Source: J Med Internet Res. 2025 Nov 24;27:e80410. doi: 10.2196/80410 (PMC12686853; doi:10.2196/80410)
Supplement: Multimedia Appendix 10 [file jmir_v27i1e80410_app10.docx]

|  | | | | | |
| --- | --- | --- | --- | --- | --- |
| β (95 CI%), P-value | Sleep duration* |  | PHQ-9 scores |  | GAD-7 scores |
| MPAI^a^ | −0.017 (−0.019 to −0.016), <.001 |  | 0.049 (0.047 to 0.051), <.001 |  | 0.207 (0.203 to 0.212),<.001 |
| Suicide scores^b^ | −0.065 (-0.072 to -0.058), <.001 |  | 0.076 (0.017 to 0.081), <.001 |  | 0.053 (0.052 to 0.056), <.001 |
| a:Multiple linear regression was used with mediating factors as the dependent variable and MPAI as the independent variable  b:Multiple linear regression was used with suicidal score as the dependent variable and mediating factors as the independent variable model was adjusted for the same variables as adjusted model in Table 2. * The model was further adjusted wake and bed lag. MPAI, mobile phone addiction index; PHQ-9, Patient Health Questionnaire 9-item scale; GAD-7, Generalised Anxiety Disorder 7-item scale. | | | | | |
